# Insights into the Antimicrobial Mechanisms of Cinnamon (*Cinnamomum verum*) and Oregano (*Origanum vulgare*) Essential Oils Against Foodborne Pathogens

**DOI:** 10.3390/molecules31173060

**Published:** 2026-08-31

**Authors:** Federica Barbieri, Chiara Montanari, Martina Filippini, Stefania Arioli, Vida Šimat, Giulia Tabanelli, Fausto Gardini

**Affiliations:** 1 Department of Agricultural and Food Sciences, University of Bologna, 47521 Cesena, Italy; federica.barbieri16@unibo.it (F.B.); martina.filippini4@unibo.it (M.F.); fausto.gardini@unibo.it (F.G.); 2 Department of Food, Environmental and Nutritional Sciences (DeFENS), Università Degli Studi di Milano, Via Celoria 2, 20133 Milan, Italy; stefania.arioli@unimi.it; 3 Faculty of Marine Sciences, University of Split, HR-21000 Split, Croatia; vida@unist.hr; 4 Department of Agricultural and Food Sciences, University of Bologna, 40127 Bologna, Italy; giulia.tabanelli2@unibo.it

**Keywords:** essential oils, antimicrobial activity, flow cytometry, viable but non-culturable (VBNC) cells, oregano, cinnamon

## Abstract

Essential oils (EOs) are promising natural antimicrobial agents, although their mechanisms of action are not yet fully understood. This study investigated the antimicrobial mechanisms of cinnamon (*Cinnamomum verum*) and oregano (*Origanum vulgare*) EOs against *Listeria monocytogenes* Scott A and *Staphylococcus aureus* DSM 20231^T^ by combining conventional plate counting with multiparameter flow cytometry. Bacterial cells were exposed to different sub-lethal or lethal EO concentrations, and culturability and three complementary physiological endpoints—viability, membrane permeability, and membrane potential—were monitored during treatment and after stress removal. Oregano EO, rich in carvacrol, rapidly reduced bacterial culturability and induced extensive membrane damage and cell death in both species. In contrast, cinnamon EO, characterized by *trans*-cinnamaldehyde as its main constituent, produced a slower antimicrobial response, with more limited membrane permeabilization and a greater recovery after stress removal. Discrepancies between culturability and flow-cytometric viability revealed transient VBNC-like populations, while post-stress recovery demonstrated that loss of culturability was reversible under some conditions. Overall, cinnamon EO-induced injury was at least partially repairable, particularly in *L. monocytogenes*, whereas damage caused by oregano EO at the highest tested concentration was essentially irreversible. These findings clarify the distinct antimicrobial effect of the two EOs and support their rational application as natural food preservatives.

## 1. Introduction

Growing interest in the antimicrobial and antioxidant properties of plant-derived compounds has stimulated extensive research on essential oils (EOs) in recent decades. More than 1300 plant species, comprising over 30,000 bioactive components, have been investigated for their antimicrobial potential [1]. Among plant-derived antimicrobials, polyphenols and EOs are particularly relevant. EOs are complex mixtures of poorly water-soluble compounds, mainly terpenes, terpenoids, and phenylpropanoids [2,3,4]. Their marked lipophilicity facilitates interaction with microbial cell membranes, which are considered the primary target of their antimicrobial activity [1,5].

Numerous EOs from officinal plants and spices have been evaluated in food systems as potential alternatives to synthetic preservatives. However, despite promising results, several limitations hinder their industrial application, including: (*i)* compositional variability affecting antimicrobial efficacy; (*ii)* organoleptic impact; (*iii)* lack of standardized assessment methods; (*iv)* limited knowledge of interactions with food matrices and processing conditions; (*v)* incomplete understanding of mechanisms of action; and (*vi)* scarce characterization of synergistic or antagonistic interactions among EO constituents [1,2,3,6,7].

Several species of the genus *Cinnamomum* (Lauraceae) are recognized for their production of EOs, which can be extracted from leaves, bark, and seeds. The most used species include *Cinnamomum verum* (syn. *C. zeylanicum*), *C. cassia* (syn. *C. aromaticum*), *C. camphora*, and *C. burmanni*. Cinnamon spice is obtained from the bark of these species [8,9], and their EOs (cassia oil and cinnamon oil) are widely applied in traditional medicine [10]. *Trans*-cinnamaldehyde, a phenylpropanoid synthesized from phenylalanine, is generally the predominant constituent of these EOs. This α,β-unsaturated aldehyde exhibits significant antimicrobial activity, mainly attributed to its reactive carbonyl moiety [9]. Other phenylalanine derivatives, such as cinnamic acid, cinnamyl acetate, and eugenol, may also be present, along with minor amounts of terpenes and terpenoids including borneol, camphor, β-caryophyllene, nerolidol, and α-terpineol. Typically, *trans*-cinnamaldehyde predominates in bark EOs, whereas eugenol is more abundant in leaf EOs [11,12,13]. The cytoplasmic membrane represents the first target of cinnamaldehyde’s antimicrobial activity. Owing to its lipophilic aromatic ring, *trans*-cinnamaldehyde can partition into the phospholipid bilayer. However, unlike many terpenes and terpenoids, it also exhibits moderate water solubility, enabling diffusion into the cytoplasm, where its reactive aldehyde group interacts with cellular components, leading to intracellular damage and potentially cell death [7,9].

The term “oregano” broadly refers to botanically distinct species depending on the geographical region, including plants primarily from the *Lamiaceae* (e.g., *Origanum, Calamintha, Hedeoma*) and *Verbenaceae* (*Lippia, Lantana*) families, as well as species from *Asteraceae* and *Fabaceae* [13]. Within the genus *Origanum*, several species, such as *O. vulgare*, O. onites, *O. compactum*, *O. majorana*, *O. bilgeri* and *O. syriacum*, have been investigated for antimicrobial activity. As with most EOs, the chemical composition of *Origanum* spp. varies considerably according to species, chemotype, agronomic and environmental conditions, harvest time, and extraction method [14,15]. Despite this variability, oregano EOs are typically dominated by terpenes and terpenoids, particularly carvacrol, thymol, and p-cymene. Other frequently detected compounds include γ-terpinene, linalool, terpinen-4-ol, β-myrcene, and β-caryophyllene [13,14]. The relative abundance of these constituents strongly influences antimicrobial efficacy in food systems. In *O. vulgare* EOs produced in Italy, carvacrol is frequently the predominant molecule, often exceeding 60% of the total composition [16,17,18,19]. The antimicrobial efficacy of carvacrol-rich EOs has been widely documented [13] and, as well as for its isomer thymol, is primarily related to its hydrophobic nature and the presence of a hydroxyl group on the aromatic ring. Following incorporation into the cytoplasmic membrane, carvacrol disrupts membrane-associated functions and acts as a proton exchanger, leading to dissipation of the proton motive force [20,21]. Thus, its antimicrobial effect is mainly associated with membrane permeabilization and depolarization [22].

Recent evidence has demonstrated significant antimicrobial activity of *C. verum* and *O. vulgare* EOs against *Listeria monocytogenes* and *Staphylococcus aureus*. For *C. verum* EO, Minimum Inhibiting Concentration (MIC) and Minimum Bactericidal Concentration (MBC) values of 250 and 500 mg/L, respectively, were reported for both strains, whereas *O. vulgare* EO exhibited MIC and MBC values of 250 and 300 mg/L, respectively. The main constituent of *C. verum* EO was *trans*-cinnamaldehyde (62%), followed by cinnamyl acetate (14.1%) and eugenol (4.5%). In *O. vulgare* EO, carvacrol (75.9%) predominated, with p-cymene (7.4%) and γ-terpinene (5.1%) as secondary components [23]. Both these EOs and their principal constituents had been previously investigated for antimicrobial activity against *L. monocytogenes* [8,22,24,25,26] and *S. aureus* [22,26,27,28].

Despite this body of evidence, the available studies remain fragmented. Most have examined individual EOs or their major constituents, often against limited sets of bacterial strains and under heterogeneous experimental conditions, thereby limiting direct comparisons across studies [8,22,24,25,26,27,28]. Moreover, the literature has largely focused on immediate antimicrobial effects during treatment, such as growth inhibition, loss of culturability, or membrane damage, while the persistence and reversibility of cellular injury after stress removal have received limited attention. Thus, a direct comparison of two chemically distinct EOs within a common experimental framework, combining culture-dependent analyses, multiparameter physiological measurements, and post-stress recovery assays, is still lacking.

To address these gaps, the innovation of this work lies in the side-by-side evaluation of oregano and cinnamon EOs, with distinct predominant constituents and modes of action under identical experimental conditions. The target microorganisms were *S. aureus* DSM 20231^T^ and *L. monocytogenes* Scott A and conventional plate counting was integrated with multiparameter FCM to monitor culturability, viability, membrane permeability, and membrane potential during EO exposure and after stress removal.

## 2. Results

### 2.1. Effect of EOs on Listeria monocytogenes Viability and Culturability

**Figure 1:**
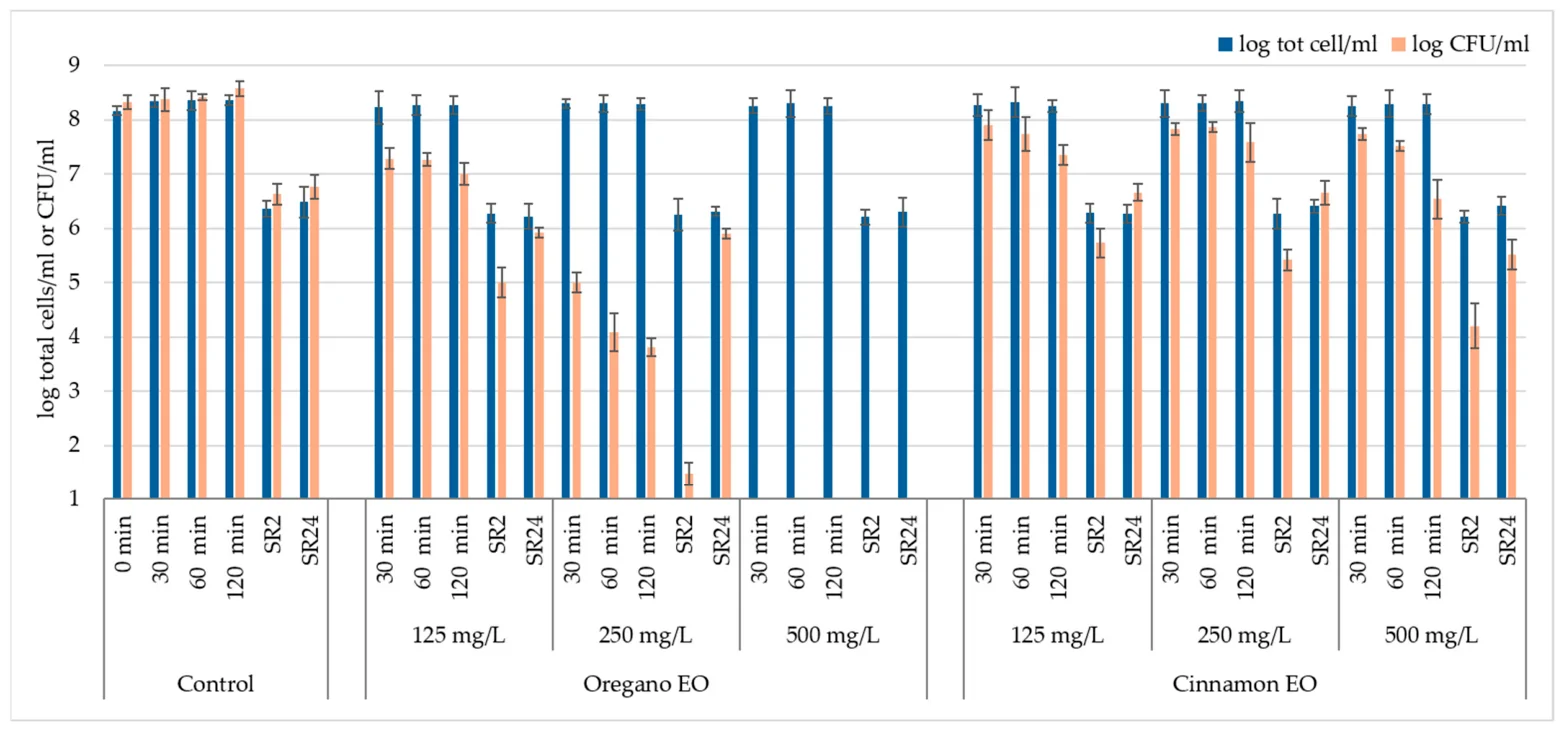
Comparison between the data of cell culturability (expressed as log CFU/mL) and the total cells detected by FCM analysis (log tot cells/mL) of *L. monocytogenes* Scott A during exposure (30, 60 and 120 min) to oregano or cinnamon EOs and after 2 (SR2) and 24 (SR24) hours from stress removal.

The effects of the different EOs on *Listeria monocytogenes* are shown in Figure 1, which compares total cell counts determined by flow cytometry (FCM), using SYBR Green I stain, with culturability values obtained by plate counting under the different experimental conditions. FCM detects the total number of cells present in the sample, regardless of their physiological state (live, injured or dead), whereas plate counts quantify only cells capable of forming colonies under the applied culture conditions, thus excluding the possible presence of viable but non-culturable (VBNC) cells. In control samples (i.e., absence of EO), no differences were observed in the different conditions. At all sampling times, cell loads exceeded 8 log CFU/mL. Similarly, no differences were detected in the control samples used as references for stress removal tests (SR2 and SR24), although microbial concentrations were lower due to the 100-fold dilution applied to remove the stress conditions (see Section 4.3). The cell exposure to oregano EO at a sub-inhibitory concentration corresponding to half the MIC (125 mg/L) produced consistent results across exposure times (30–120 min). Total cell counts determined by FCM were comparable to those of the untreated control, whereas culturability decreased by approximately 1 log unit (≈90%). This discrepancy persisted after 2 h from stress removal, indicating the presence of cells with impaired culturability. After 24 h (SR24), however, FCM and plate count values converged, suggesting restoration of culturability and repair of cellular damage. A different trend was observed at the MIC concentration (250 mg/L). Compared with FCM counts, culturability decreased by approximately 3 log CFU/mL after 30 min of exposure and continued to decline with increasing contact time. After 120 min, the difference exceeded 4 log units. Following stress removal, a further reduction was observed after 2 h (SR2) because of sample dilution, whereas after 24 h (SR24) the values obtained by the two methods converged. At a concentration twice the MIC (500 mg/L), the number of events detected by FCM remained constant, whereas culturability fell below the detection limit (<1 log CFU/mL) both in exposed samples and during the subsequent stress removal tests.

The physiological state of cells exposed to different EOs was further investigated by dual-staining FCM analysis, to identify subpopulations of live, injured, and dead cells based on cell membrane permeability to propidium iodide. The results are summarized in Table 1. In the control (i.e., absence of EOs), more than 95% of cells were live at all sampling times, whereas damaged and dead cells together accounted for less than 5% of the total population. At half MIC (125 mg/L) of oregano, dead cells accounted for approximately 62% of the total population only after 2 h of exposure. Conversely, live cells represented only 10% of the population after 2 h and further decreased as the EO contact time increased. A slight increase in live cells (about 15%) was observed only after 24 h of the stress-removal phase. Treatment at the MIC (250 mg/L) resulted in a marked increase in dead cells, which represented more than 95% of the population. After 24 h from the stress removal, the percentage of live/injured cells slightly increased; however, dead cells still accounted for more than 85% of the population. Exposure to 500 mg/L oregano EO was lethal to *L. monocytogenes*, and only dead cells were detected under all tested conditions, consistent with the plate count results.

When *L. monocytogenes* Scott A was exposed to cinnamon EO, the comparison of the data of cell culturability (log CFU/mL) and the total cells detected by FCM analysis (Figure 1) showed that the presence of 125 mg/L of this EO resulted in an approximately 1 log reduction in culturability after 2 h of treatment. However, after 24 h from stress removal, live counts returned to levels comparable to those of the control. A similar trend was observed at the MIC values (250 mg/L). Treatment with 500 mg/L EO (corresponding to MBC) caused a more pronounced reduction in culturability after 2 h of exposure (greater than 2 log units), while FCM counts remained unchanged, indicating the absence of cell lysis. No significant changes were observed after 2 h from stress removal, whereas after 24 h the counts returned to levels comparable to the control, suggesting a restored culturability. However, cell proliferation cannot be excluded on the basis of FCM and plate-count data alone.

Concerning the viability of *L. monocytogenes* cells in the presence of cinnamon EO (Table 1), the concentration corresponding to half MIC (125 mg/L) resulted in cell mortality ranging from 30% to 50%, increasing with exposure time and accompanied by a decrease in live cells, while the proportion of damaged cells remained relatively constant. After removal of stress, no substantial differences were observed after 2 h, while, after 24 h, the proportion of live cells increased, reaching approximately 40% of the total population. At the MIC concentration (250 mg/L), a further reduction in live cells was observed, whereas the proportion of dead cells remained relatively stable. After 24 h from stress removal, the percentage of live cells was significantly lower than that observed at the lower EO concentration, accounting for about 18% of the total bacterial population. Treatment with 500 mg/L EO caused a marked increase in cell mortality and a drastic reduction in live cells with increasing exposure time; dead cells exceeded 80% after 2 h of exposure. After 24 h from stress removal, partial restoration of viability was observed (approximately 16% of the total population).

### 2.2. Effect of EOs on Staphylococcus aureus Viability and Culturability

**Figure 2:**
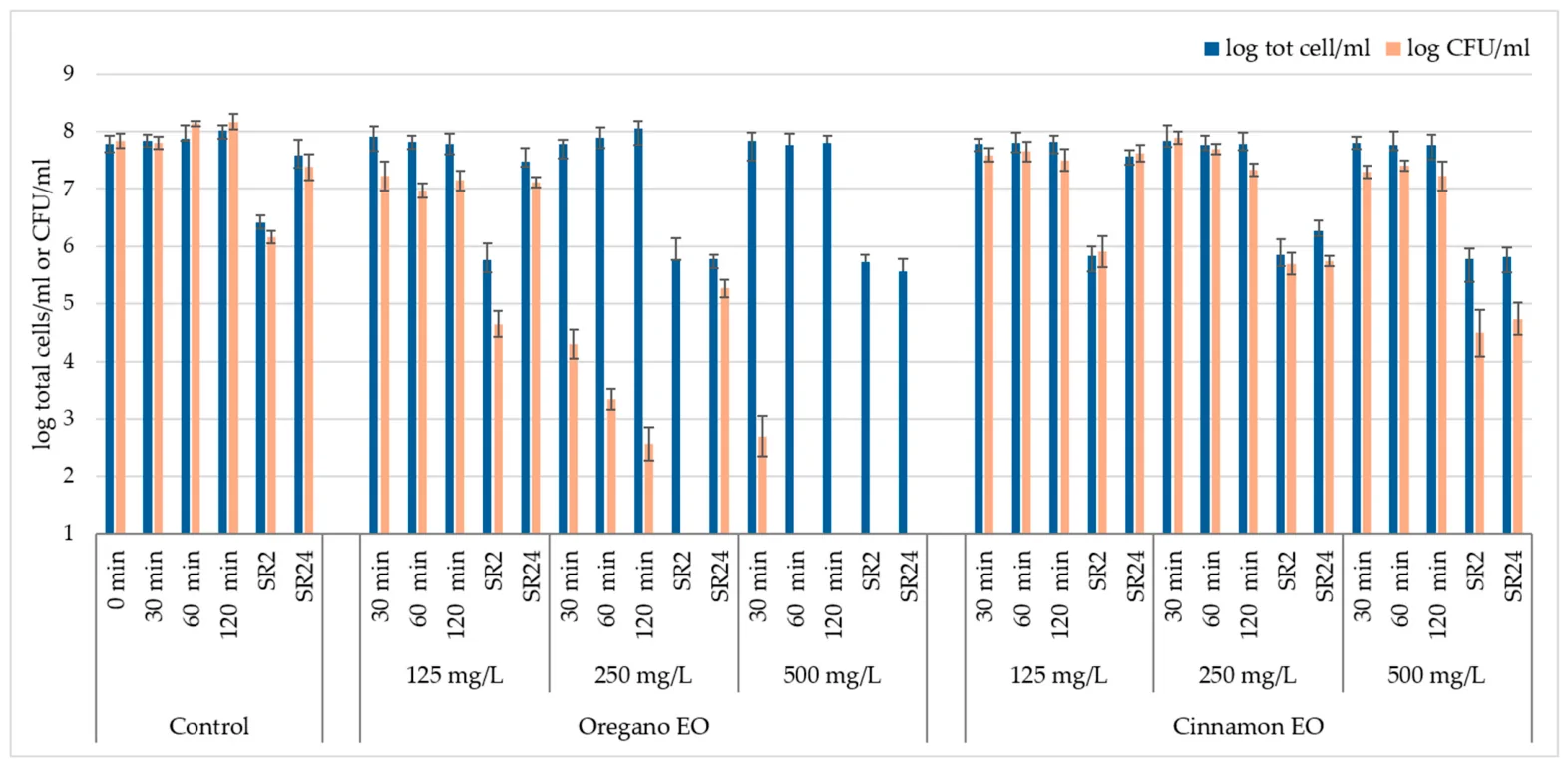
Comparison between the data of cell culturability (expressed as log CFU/mL) and the total cells detected by FCM analysis (log total cells/mL) of *S. aureus* DSM 20231^T^ during exposure (30, 60 and 120 min) to oregano or cinnamon EOs and after 2 (SR2) and 24 (SR24) hours from stress removal.

Comparative data between plate counts and FCM events for *S. aureus* after exposure to the different EOs are shown in Figure 2. As observed for *L. monocytogenes*, microbial populations determined by the two methods were comparable in all control samples, and no substantial differences were detected. In addition, during the recovery test, cells were able to grow by approximately 1 log cycle within 24 h. Exposure to oregano EO at 125 mg/L resulted in an approximately 1 log CFU/mL reduction in culturability, regardless of treatment time. This discrepancy between plate counts and FCM data persisted after 2 h from stress removal. However, after 24 h, the number of total cells increased by about 1.5 log cycles, suggesting cell multiplication, and culturability was totally restored, as indicated by the convergence of the two measurements.

At the MIC value (250 mg/L), a marked reduction in culturability was observed, increasing with exposure time. Indeed, reductions exceeded 4 log cycle units already after 30 min, and reached approximately 5.5 log cycles after 120 min. After 2 h from stress removal, culturability remained below the detection limit, whereas after 24 h an almost complete restoration of colony-forming ability was observed. In contrast, exposure to oregano EO at twice the MIC (500 mg/L) produced a rapid antimicrobial effect. After 30 min, plate counts decreased to below 3 log CFU/mL, and no culturable cells were detected at later sampling times or after stress removal, indicating irreversible loss of culturability under these conditions.

The FCM analysis of *S. aureus* treated with oregano or cinnamon EOs (Table 2) revealed that in the control samples more than 90% of cells remained alive at all sampling times. When oregano EO was added at half MIC (125 mg/L), 40–60% of cells were injured or dead. After 24 h from stress removal, the proportion of live cells increased to more than 98% of the total population, consistent with plate count results. Treatment with oregano EO at the MIC value (250 mg/L) resulted in more than 95% dead cells during the exposure phase. After stress removal, the proportion of live cells was constant after 2 h and then increased, reaching more than 26% of the total population after 24 h. Finally, at 500 mg/L, under all tested conditions, almost all cells (>99% of the total population) resulted dead.

The data obtained for *S. aureus* exposed to cinnamon EO (Figure 2) showed that the total cell counts determined by FCM and plate counting were comparable at EO concentrations up to 250 mg/L (MIC). After 2 h of exposure at the MIC, a slight reduction in culturability (<1 log unit) was observed, and this difference persisted also after stress removal. At 500 mg/L (MBC), plate counts were lower (less than 1 log cycle) and, after stress removal (2 or 24 h), a more pronounced decline in culturability (about 1.5 log cycle) was observed. Concerning the effect of cinnamon EO on the physiological state of *S. aureus* (Table 2), in samples treated with 125 mg/L, the proportion of live cells remained high (60–70%) across all exposure times and after 2 h from stress removal. Moreover, after 24 h, almost all cells (about 98%) showed restored viability. At the MIC (250 mg/L), the percentage of live cells was initially around 70%, with a very low proportion of dead cells (less than 3%). However, after EO stress removal, the proportion of live cells progressively decreased to approximately 30% after 24 h, accompanied by a marked increase in dead cells (about 53%). Similar trends were observed at 500 mg/L EO (MBC), even if live cells after 24 h from stress removal accounted for about 10% of the total population, with a higher occurrence of injured cells. Overall, the ability of the species to restore cell functionality after stress removal decreased at EO concentrations of 250 mg/L or higher.

### 2.3. Effect of EOs Exposure on Membrane Depolarization

To better understand the action mechanism of the two EOs against the target pathogens, the modifications of cell membrane potential during exposure to EOs were monitored through DiBAC_4_ staining of treated cells. An increase in green fluorescence indicated cell depolarization, i.e., a reduction of the proton motive force (PMF) across the plasma membrane. Gramicidin A, an antimicrobial peptide known to induce dissipation of the electrochemical gradient across the cell membrane [29], was used as a positive control. The data are summarized in Table 3, together with information about membrane permeability (PI fluorescence) and percentage of dead cells (obtained with dual staining SYBR Green I and PI). Since membrane depolarization is a rapid and dynamic reaction that can precede harsher damages, concentrations up to MIC values (250 mg/L) were only tested, and cells were monitored for 120 min.

Concerning *L. monocytogenes*, DiBAC_4_ uptake was low and constant in control cells, while the presence of oregano EO induced an increase in fluorescence, thus a depolarization effect related to the EOs concentration. These reactions occurred in the first 30 min of exposure and then values remained almost stable during the following sampling times. As far as membrane permeability, a notable increase in PI uptake, occurring within the first 30 min of exposure, suggested concomitant membrane damage, even if the percentage of dead cells differed in relation to the amount of the EOs. The presence of cinnamon EO determined higher cell membrane depolarization, with values doubled with respect to oregano EO, while PI uptake was lower, suggesting that membrane integrity was less affected. Indeed, the mortality rate reached approximately 45% after 120 min of cinnamon EO exposure at 250 mg/L.

*S. aureus* was found to be more sensitive to membrane depolarization in the presence of oregano, with values almost doubled with respect to *L. monocytogenes*, independently of EO amount. Data on PI uptake showed that membrane integrity was strongly affected by 250 mg/L of oregano EO, with almost the entire population (>95%) recognized as dead. Cinnamon EO determined similar values of DiBAC_4_ uptake, but lower PI fluorescence, corresponding also to mortality rates less than 3%. Interestingly, for both EOs at half MIC values (125 mg/L), a decrease in DiBAC_4_ fluorescence, corresponding to a reduction in membrane depolarization, was observed throughout 120 min of exposure, suggesting an active response from *S. aureus* in counteracting EOs effects when present at sublethal concentrations.

## 3. Discussion

The two EOs investigated in this study exhibited distinct antimicrobial effects against the tested bacteria. Although both showed the same MIC (250 mg/L), the physiological responses of the cells differed. After 2 h of exposure to 250 mg/L EOs, cells treated with oregano EO showed greater damage, both in terms of culturability and physiological state, compared with those treated with cinnamon EO. At 500 mg/L, oregano EO exerted a rapid bactericidal effect against both species, while this condition was not completely verified with cinnamon EO after the same time. Differences between species were also observed, with *S. aureus* showing greater resistance to EO-induced stress than *L. monocytogenes*. The discrepancies between live and culturable cells evidenced the possible occurrence of VBNC cells. FCM analysis indicates that these differences cannot always be explained simply by the presence of live or dead cells. The fate of injured cells remains difficult to predict, as shown by stress removal tests, and in particular the viability recovery observed after 24 h in *L. monocytogenes* previously exposed to 500 mg/L of cinnamon EO. Similar responses have been reported for *L. monocytogenes* subjected to mild thermal or terpene stresses [30,31]. The results also suggest that cinnamon EO requires longer exposure times to exert its antimicrobial and especially bactericidal effect. After 2 h of exposure, only a small proportion of cells exhibited severe damage at MIC (250 mg/L), whereas oregano EO caused marked reductions in both culturability and viability. Consistently, previous studies reported that more than 90% of *L. monocytogenes* cells showed membrane damage after 24 h exposure to carvacrol in buffer at pH 7 and 4 [22]. Moreover, carvacrol and other terpene molecules often exert a marked bactericidal effect at concentrations very close to the MIC, as indicated by the absence of colony formation and by the small difference generally observed between MIC and MBC values [20,23].

The antimicrobial activity of carvacrol, the principal component of oregano EO, is mainly associated with the presence of a free hydroxyl group and a phenolic ring enabling electron delocalization [20,21]. Due to its lipophilic nature (water solubility 0.11 g/L), carvacrol partitions into cell membranes, rather than penetrating the aqueous cytoplasmic environment, increasing membrane fluidity and permeability [5,32]. In *L. monocytogenes*, an increase in the proportion of unsaturated fatty acids and a decrease in branched fatty acids were observed as a response to membrane fluidity perturbation [32]. Concerning *S. aureus*, the presence of carvacrol increased the ratio between unbranched and branched fatty acids [33]. In addition, the hydroxyl group acts as a proton exchanger, reducing the transmembrane gradient and collapsing the PMF [20,21].

Regarding cinnamon EO, previous studies have reported that *trans*-cinnamaldehyde exerts its antimicrobial activity mainly through intracellular targets [34,35,36]. Indeed, the main effects observed in *S. aureus* include potassium ion leakage, reduced metabolic activity, and impaired replication. Membrane integrity does not appear to be the primary target, since exposure of *S. aureus* to cinnamon EO at the MIC did not markedly affect membrane permeability [27], with no substantial PI uptake, as also observed in the present study. This behavior may be related to the physicochemical properties of *trans*-cinnamaldehyde, which, after partitioning into the bacterial membrane, may diffuse into the aqueous cytoplasmic environment inactivating enzymes and interacting with other essential cellular molecules [24,37]. This mechanism may also explain why only limited damage was observed after 2 h of exposure at the MIC, as indicated by both FCM-based viability assessment and plate-count culturability data. In *L. monocytogenes*, the antimicrobial action of *trans*-cinnamaldehyde interferes with energy metabolism, causing a decrease in cellular ATP levels and suggesting dissipation of the PMF through membrane perturbation and leakage of small ions [38].

The results concerning membrane depolarization and permeabilization confirmed differences in the action mechanism of the two EOs, as well as in the response of the two target strains. While PI uptake is possible only in the presence of significant membrane damage, DiBAC_4_ uptake may occur even in less-damaged cells [39], and a strong disruption of membrane integrity can even hinder its binding to the cell target [40]. Bacterial membrane potential is increasingly recognized as a dynamic physiological parameter involved not only in energy conservation, but also in stress responses (e.g., antibiotic resistance), cellular adaptation, and electrochemical signaling [41]. Therefore, depolarization generally reflects the dissipation of ion gradients and PMF, often associated with membrane malfunctioning or impaired energy metabolism. By contrast, an irreversibly dead cell is not expected to maintain a functional membrane potential for long [42]. The findings of the present study are consistent with previous evidence indicating that, for several monoterpenoids and phenylpropanoids occurring in EOs, membrane depolarization can precede the increase in membrane permeability that reflects more severe loss of cell integrity. For example, some Authors showed that carvacrol at MIC induced a rapid depolarization of *S. aureus* cells within 5 min of exposure, while membrane permeabilization remained comparatively limited during the same period [43]. Conversely, and in contrast with the observations reported here, Bouhdid et al. [27] found no or only negligible changes in *S. aureus* membrane potential after 60 min of exposure to cinnamon EO at the MIC. Thus, depolarization and permeabilization do not necessarily correlate, and depolarization does not necessarily coincide with cell damage. Indeed, damaged bacterial cells may, under some conditions, retain measurable membrane potential; for example, Novo et al. [44] reported subpopulations with increased membrane permeability but physiological membrane potential, highlighting that membrane integrity and membrane potential are not necessarily equivalent readouts.

In the present study, *L. monocytogenes* was less prone to staining with DiBAC_4_, showing lower fluorescence values than *S. aureus*. Although this could be explained with an ability to maintain membrane potential, the data on membrane permeability, especially for oregano EO, seem to indicate severe damage to membrane integrity, which can affect the ability of DiBAC_4_ to bind the cell. Indeed, previous studies obtained with the same strain in the presence of terpenoids had shown a decrease in DiBAC_4_ fluorescence in the harder stress conditions [45]. Conversely, *S. aureus* exhibited higher DiBAC_4_ uptake and, in general, lower PI fluorescence. This can be due to less severe damage induced by the two EOs, except for oregano at MIC level. In addition, the decrease in DiBAC_4_ values during exposure at half MIC concentration of EOs suggested an active response of the cells in restoring the membrane potential.

These different behaviors have direct implications for food preservation and hurdle technology design. The consequences of a 2-h exposure of the cells at EOs concentration corresponding to MBC should not automatically be interpreted as evidence of irreversible inactivation: with cinnamon EO, a reduction in antimicrobial pressure caused by dilution may permit injured or VBNC-like cells to recover. Consequently, cinnamon EO may require longer contact times or combination with additional, persistent hurdles, whereas oregano EO at 500 mg/L produced a more durable bactericidal outcome under the present experimental conditions. It has recently been reported that, when applied to real matrices, other variables can be involved, such as food composition, that can potentially affect antimicrobial activity [46].

Taken together, the use of a common experimental framework enabled a direct comparison of two EOs characterized by distinct predominant constituents and modes of action, addressing the heterogeneity of earlier studies focused on individual oils. However, the application of EOs should also account for a natural batch-to-batch compositional variability, in relation to geographical origin, season, and extraction methods [3], affecting their effectiveness as antimicrobials. In the present study the EOs were commercial products, and the same lots were used for all the experiments, to allow reproducibility. With the final aim of exploiting these natural antimicrobials in real systems, the results obtained with these single EOs should also be validated in other EOs extracted from the same plants.

Monitoring culturability, viability, membrane permeability, and membrane potential both during treatment and after stress removal further showed that cellular fate cannot be inferred from immediate antimicrobial effects alone.

## 4. Materials and Methods

### 4.1. Bacterial Strains and Growth Conditions

The strains used in this study were *Listeria monocytogenes* Scott A and *Staphylococcus aureus* DSM 20231^T^ belonging to the collection of the Department of Agricultural and Food Sciences (University of Bologna, Bologna, Italy). *L. monocytogenes* Scott A was chosen because widely used as a reference strain to test the efficacy of food processing and preservation techniques, as well in growth and heat resistance studies [47], while *S. aureus* DSM 20231^T^ is the type strain for the species. Both strains were maintained in BHI medium (Oxoid, Basingstoke, UK) with 30% (*w*/*v*) glycerol at −80 °C and, before the experiments, pre-cultivated twice (37 °C for 24 h) in BHI medium.

### 4.2. Essential Oils

Oregano (*Origanum vulgare* L.) EO obtained from flower parts and cinnamon (*Cinnamomum verum*) EO obtained from bark were purchased from Flora srl (Pisa, Italy) and were previously characterized for their composition and antimicrobial activity [23]. A total of 26 and 30 compounds were identified in oregano and cinnamon EO, respectively, and the main constituents grouped into chemical classes with their relative abundance are summarized in Table 4. EOs were dissolved in absolute ethanol (Sigma-Aldrich, Milan, Italy) to prepare proper solutions, that were added to the different samples without exceeding 0.5% *v/v* as final concentration of ethanol, to avoid possible antimicrobial activity due to its presence.

### 4.3. Effect of Oregano and Cinnamon EOs on Listeria monocytogenes and Staphylococcus aureus

To assess the effect of sub-lethal or lethal concentrations of the two EOs, the target strains were inoculated in sodium phosphate buffer (PBS, pH 7.4, Dulbecco A Oxoid, Thermo Fisher Diagnostics, Milan, Italy), added with 0.1% (*w*/*w*) of bacteriological peptone (Oxoid, Thermo Fisher Diagnostics, Milan, Italy), at a cell load of approx. 8 log cell/mL in the presence of different concentrations EOs: 0 (control), 125 (half of MIC value), 250 (MIC value) and 500 (double of MIC value) mg/L. These amounts were chosen based on previous trials carried out to assess the antimicrobial activity of several commercial EOs [23]. Since EOs were dissolved in ethanol, a control without EOs was added with the same amounts of ethanol (0.5% *v/v* in the final medium). Samples were incubated at 37 ± 0.5 °C and collected after 30, 60 and 120 min to be analysed both by culture-dependent method (plate counting) and culture-independent (flow cytometry) methods. Then, to remove stress conditions due to EO exposure and to assess a potential recovery of treated cells, the samples were diluted 1:100 in PBS added with peptone 0.1% and re-incubated at 37 °C, to be analysed after 2 (SR2) and 24 (SR24) hours. The analyses were performed in triplicate.

### 4.4. Plate Counting

To assess culturability, samples were collected at each sampling time, and 0.1 mL of appropriate decimal dilutions were plated onto BHI agar medium (Oxoid, Thermo Fisher Diagnostics, Milan, Italy). Plates were incubated at 37 °C for 48 h. The detection limit was 1 log CFU/mL.

### 4.5. Flow Cytometry (FCM) Analysis

At each sampling time, cell suspensions derived from the different conditions were analysed through flow cytometry to assess viability, membrane permeabilization and membrane depolarization. A flow cytometer Accuri C6 (BD Biosciences, Milan, Italy) was used with the following threshold settings: FSC 3000 and SSC 1000, 30,000 total events collected, medium flow rate. Before analyses, samples were diluted (if needed) in filtered PBS and the cells were stained in the dark at 37 °C for 15 min with different fluorochrome, all purchased from Sigma-Aldrich (Milan, Italy): SYBR-Green I (1×) for the total cell count, SYBR-Green I (1×) and propidium iodide (PI) 7.5 μM in combination, to discriminate three sub-populations corresponding to live, injured, dead cells and to monitor membrane permeability, and Bis-(1,3-Dibutylbarbituric Acid) Trimethine Oxonol (DiBAC_4_) 3.0 μM to assess membrane depolarization [45]. The SYBR-Green I and DiBAC_4_ fluorescence intensities of stained cells were recovered in the FL1 channel (excitation 488 nm, emission filter 530/30 nm). The PI fluorescence (excitation 488 nm, emission filter 630/30 nm) intensity of stained cells was recovered in the FL3 channel. The data obtained were analysed using the BD ACCURITM C6 software version 1.0 (BD Biosciences, Milan, Italy).

## 5. Conclusions

The distinctive contribution of this study lies in integrating plate counting with multiparameter FCM, assessing different cell membrane-related parameters, during EOs exposure and after stress removal. Our approach was applied to characterize the effect of these natural antimicrobials against two foodborne pathogens, highlighting different behaviors. Moreover, our findings suggested that, after stress removal, the loss of culturability associated with transient VBNC-like states may be reversible. Cinnamon EO-induced injury at 500 mg/L was partially repaired, after stress removal, within 24 h, particularly by *L. monocytogenes*, whereas oregano EO caused essentially irreversible damage.

While this study was performed in a model system, our results could provide a baseline for understanding of EOs mechanisms. Future research should expand these findings by testing a wider range of bacterial species and strains, also evaluating the variability of EOs composition, and investigating combined intracellular stresses, such as oxidative stress. Scaling up this approach into food matrices is an essential step to determine how food constituents affect EOs antimicrobial efficacy and to validate cell recovery potential in real systems.

## Figures and Tables

**Table 1:** Percentage distribution of live, injured and dead cells of *L. monocytogenes* Scott A during exposure (30, 60 and 120 min) to oregano or cinnamon EOs and after 2 (SR2) and 24 (SR24) hours from stress removal. The data are reported as the relative frequency of the total population obtained by FCM analysis with dual staining (SYBR-Green I and PI).

Condition		Time	Live	Injured	Dead
Control		0 min	94.91	3.85	1.24
		30 min	95.30	2.37	2.32
		60 min	94.55	2.33	3.12
		120 min	94.88	2.56	2.56
		SR2	94.86	1.94	3.20
		SR24	96.83	0.80	2.37
Oregano EO	125 mg/L	30 min	10.19	24.76	65.05
		60 min	8.35	31.70	59.96
		120 min	7.38	30.31	62.31
		SR2	5.85	25.69	68.46
		SR24	15.46	19.37	65.17
	250 mg/L	30 min	0.37	2.99	96.64
		60 min	1.14	1.14	97.72
		120 min	1.14	1.25	97.61
		SR2	5.71	0.73	93.55
		SR24	5.75	8.25	86.00
	500 mg/L	30 min	0.31	0.41	99.28
		60 min	0.17	0.23	99.60
		120 min	0.18	0.47	99.36
		SR2	0.17	0.22	99.61
		SR24	0.07	0.14	99.79
Cinnamon EO	125 mg/L	30 min	35.70	32.30	32.00
		60 min	27.94	29.99	42.07
		120 min	18.35	34.03	47.62
		SR2	25.69	29.57	44.74
		SR24	40.34	12.61	47.05
	250 mg/L	30 min	24.23	36.28	39.49
		60 min	15.83	35.95	48.22
		120 min	16.19	38.38	45.44
		SR2	11.00	23.73	65.27
		SR24	18.43	17.47	64.10
	500 mg/L	30 min	18.12	37.01	44.86
		60 min	8.18	33.97	57.85
		120 min	2.03	17.22	80.75
		SR2	1.52	8.18	90.30
		SR24	16.12	18.46	65.43

**Table 2:** Percentage distribution of live, injured and dead cells of *S. aureus* DSM 20231^T^ during exposure (30, 60 and 120 min) to oregano or cinnamon EOs and after 2 (SR2) and 24 (SR24) hours from stress removal. The data are reported as the relative frequency of the total population obtained by FCM analysis with dual staining (SYBR-Green I and PI).

Condition		Time	Live	Injured	Dead
Control		0 min	89.65	9.54	0.82
		30 min	91.56	7.21	1.22
		60 min	91.23	7.35	1.42
		120 min	91.63	5.05	3.33
		SR2	96.00	1.90	2.10
		SR24	97.23	2.14	0.63
Oregano EO	125 mg/L	30 min	40.93	52.58	6.49
		60 min	55.02	40.69	4.28
		120 min	47.10	47.76	5.14
		SR2	41.22	51.34	7.44
		SR24	98.80	0.73	0.47
	250 mg/L	30 min	1.10	3.45	95.45
		60 min	0.82	1.96	97.22
		120 min	0.77	0.92	98.32
		SR2	0.78	1.37	97.85
		SR24	26.27	1.40	72.33
	500 mg/L	30 min	0.00	0.34	99.66
		60 min	0.16	0.16	99.68
		120 min	0.15	0.15	99.69
		SR2	0.00	0.20	99.80
		SR24	0.61	0.31	99.08
Cinnamon EO	125 mg/L	30 min	70.46	28.12	1.42
		60 min	68.87	28.97	2.15
		120 min	69.31	28.59	2.10
		SR2	63.00	32.96	4.04
		SR24	98.16	1.25	0.59
	250 mg/L	30 min	70.05	28.37	1.58
		60 min	76.03	22.50	1.47
		120 min	67.25	30.03	2.72
		SR2	51.99	40.18	7.83
		SR24	29.91	17.07	53.02
	500 mg/L	30 min	65.98	32.14	1.88
		60 min	75.92	22.31	1.77
		120 min	46.02	48.57	5.41
		SR2	30.57	54.96	14.47
		SR24	9.47	36.65	53.88

**Table 3:** FCM analysis of *L. monocytogenes* Scott A and *S. aureus* DSM 20231^T^ during exposure to oregano or cinnamon EOs. DiBAC_4_ fluorescence and PI fluorescence values are reported as arbitrary units (AU), while the percentage of dead cells was obtained with dual staining (SYBR-Green I and PI). Data are the means of three replicates (standard deviation are reported in brackets).

Condition		Time	*L. monocytogenes*			*S. aureus*		
			DiBAC_4_ Fluorescence	PI Fluorescence	% Dead Cells	DiBAC_4_ Fluorescence	PI Fluorescence	% Dead Cells
Control		0 min	2798 (±140)	6763 (±318)	1.24 (±0.06)	11,515 (±553)	14,304 (±730)	0.82 (±0.02)
		30 min	2038 (±122)	6780 (±353)	2.32 (±0.10)	9652 (±396)	16,428 (±789)	1.22 (±0.04)
		60 min	1549 (±60)	6527 (±255)	3.12 (±0.12)	9012 (±424)	11,317 (±475)	1.42 (±0.06)
		120 min	1478 (±61)	6489 (±370)	2.56 (±0.14)	3468 (±173)	13,459 (±713)	3.33 (±0.15)
Oregano EO	125 mg/L	0 min	6176 (±303)	35,467 (±2483)	65.05 (±3.77)	32,979 (±2078)	35,412 (±2160)	6.49 (±0.15)
		30 min	6558 (±308)	35,350 (±2192)	59.96 (±2.22)	20,013 (±861)	25,404 (±1067)	4.28 (±0.10)
		60 min	7063 (±431)	23,880 (±1242)	62.31 (±2.55)	13,387 (±669)	27,432 (±1399)	5.14 (±0.14)
	250 mg/L	0 min	11,649 (±559)	38,557 (±2121)	96.64 (±2.90)	27,847 (±1420)	69,728 (±3905)	95.45 (±2.96)
		30 min	9616 (±529)	36,337 (±1526)	97.72 (±1.95)	27,811 (±1530)	63,742 (±2677)	97.22 (±2.92)
		60 min	10,763 (±495)	30,831 (±1696)	97.61 (±2.73)	22,410 (±1143)	51,302 (±2770)	98.32 (±2.75)
Cinnamon EO	125 mg/L	0 min	21,903 (±1314)	28,902 (±1474)	32.00 (±0.77)	36,692 (±1908)	25,208 (±1336)	1.42 (±0.07)
		30 min	22,255 (±1424)	22,619 (±1040)	42.07 (±1.68)	20,801 (±1061)	24,277 (±1068)	2.15 (±0.09)
		60 min	20,882 (±1169)	24,542 (±1473)	47.62 (±2.29)	8471 (±390)	24,710 (±1384)	2.10 (±0.07)
	250 mg/L	0 min	16,089 (±949)	29,681 (±1247)	39.49 (±1.86)	30,210 (±1722)	23,777 (±1308)	1.58 (±0.05)
		30 min	19,217 (±999)	23,594 (±1203)	48.22 (±2.46)	31,801 (±1749)	18,670 (±915)	1.47 (±0.04)
		60 min	16,733 (±887)	36,003 (±1728)	45.44 (±1.77)	28,232 (±1637)	19,418 (±990)	2.72 (±0.07)

**Table 4:** Chemical composition of cinnamon and oregano EOs, adapted from Barbieri et al. [23], expressed as relative percentages of each compound peak area with respect to the total peak area. Only compounds detected in amounts higher than 0.5% are reported.

Compound (%)	Cinnamon	Oregano
α-phellandrene	0.65	0.97
α-pinene	1.14	0.68
Camphene	0.57	0.08
β-pinene	0.36	0.22
β-phellandrene	2.03	0.13
β-myrcene	0.05	0.98
4-carene	0.52	0.98
p-cymene	1.41	7.36
D-limonene	0.75	0.16
γ-terpinene	0.09	5.09
**Monoterpenes**	**7.57**	**16.64**
Eucalyptol	0.27	0.07
α-terpineol	- *	0.22
Linalool	1.42	0.95
Camphor	0.69	0.03
α-terpineol	0.63	0.10
endo-borneol	0.13	0.26
Terpinen-4-ol	0.35	0.58
Thymol	-	2.66
Carvacrol	-	75.93
**Monoterpenoids**	**3.48**	**80.79**
Caryophyllene	3.01	1.38
Humulene	0.67	0.11
α-amorphene	0.75	-
**Sesquiterpenes**	**4.43**	**1.49**
trans-cinnamaldehyde	62.02	-
Eugenol	4.48	-
Acetic acid, cinnamyl ester	14.09	-
Benzyl benzoate	1.07	-
**Phenylpropanoids**	**81.66**	**-**
**Total compounds**	**97.14**	**98.92**

*: not detected.

## Data Availability

The data presented in this study are available on request from the Corresponding author.
